# Applications of Stem Cells and Modern Toxicological Analytical Methods in the Toxicity of Microplastics

**DOI:** 10.3390/toxics14070545

**Published:** 2026-06-23

**Authors:** Mohan Wang, Dilixiati Wubuli, Mulati Julaiti, Pengfei Huang, Jinghui Xie, Bowen Hu

**Affiliations:** 1 State Key Laboratory of Pathogenesis, Prevention and Treatment of High Incidence Diseases in Central Asia, Xinjiang Key Laboratory of Molecular Biology for Endemic Diseases, Department of Biochemistry and Molecular Biology, School of Basic Medical Sciences, Xinjiang Medical University, Urumqi 830017, China; mohanwang1013@163.com (M.W.); mulatijulaiti@163.com (M.J.); hpf123690@163.com (P.H.); 2 Physiology, School of Basic Medical Sciences, Xinjiang Medical University, Urumqi 830017, China; dlxtwbl@163.com

**Keywords:** microplastics, developmental toxicity, stem cell models, toxicological approaches

## Abstract

Microplastics (MPs) are an emerging environmental pollutant, and their contamination has emerged as a pressing global environmental concern. Developmental processes exhibit heightened sensitivity to environmental perturbations, and MP exposure can induce long-term adverse effects on organismal health by disrupting fundamental cellular processes. This review focuses on the toxicity and developmental toxicity risks of microplastics investigated using stem cell models. The core section comprehensively summarizes the primary mechanisms through which MP exposure interferes with the biological functions of stem cells, including the impairment of self-renewal capacity and lineage specification, as well as the inhibition of tissue-specific differentiation and organogenesis. Finally, the integration and application of modern toxicological methods in deepening research and improving risk assessment capabilities are synthesized. This review aims to provide a systematic perspective to understand the developmental hazards of MPs and look forward to future risk studies based on stem cell modeling, providing theoretical basis and fundamental support.

## 1. Introduction

Plastic products are ubiquitous. However, the pervasive environmental presence and accumulation of microplastics (MPs), an emerging class of pollutants, not only pose a potential threat to ecosystems, but also raise significant health concerns. According to literature, the global annual plastic production is approximately 400 million tons [1], while cumulative microplastic pollution in the environment is projected to reach 1.2 billion tons by 2060 [2]. This is due to their capacity for bioaccumulation and trophic transfer through food chain, ultimately leading to human exposure and potential far-reaching impacts on human health [3,4].

The toxicological mechanisms of MPs are highly complex, with their developmental toxicity being modulated by multiple factors. Key parameters, including particle characteristics, polymer type, exposure concentration, and exposure duration, have been demonstrated to significantly influence their biological effects [3]. Smaller-sized MPs are more easily taken up by organisms and can penetrate tissue barriers, thereby significantly affecting their intake and distribution within the body [5]; meanwhile, the presence of bacterial biofilms on the surface of MPs can alter their surface hydrophobicity, sedimentation behavior, and pollutant adsorption capacity, thereby enhancing their ecological toxicity potential as pollutant carriers [6], and additives in MPs (such as bisphenols and phthalates, which are endocrine disruptors) can leach from the plastic matrix, directly interfering with the biological endocrine system and exacerbating developmental toxicity [7]. Understanding the developmental toxicity of MPs is critical, as developmental stages exhibit heightened sensitivity to environmental pollutants, where even minor perturbations can induce long-term health consequences [8]. In addition, the combined toxic effects of MPs and co-existing contaminants further complicate risk assessment [9]. Despite considerable focus on the ecological risks of MPs, investigations into the specific mechanisms underlying their developmental toxicity remain limited, and direct evidence of developmental toxicity is insufficient [10], underscoring the need for effective interventions.

MPs have been shown to affect organisms during developmental stages ranging from embryogenesis and organogenesis to juvenile and fetal phases, growth inhibition, functional impairment, and a decreased survival rate [11,12]. Studies have confirmed that aged polystyrene microplastics (aged PS-MPs) are toxic to the early development of zebrafish, and can induce mitochondrial dysfunction and oxidative stress during the early life stages of zebrafish, leading to cell apoptosis [12]. Current evidence indicates that MPs can perturb the development of multiple organ systems—such as the nervous, hepatic, cardiac, and reproductive systems—through diverse mechanisms. These multisystemic adverse effects underscore the necessity for comprehensive investigation into the developmental toxicity of MPs [13]. By integrating modern toxicological methodologies such as transcriptomics and molecular dynamics simulations, these models facilitate not only the simulation of physiological effects following MP exposure but also the elucidation of underlying toxicity mechanisms. The application of such integrated approaches provides a powerful means to reveal the complex toxicology of MPs and to refine toxicity assessment strategies [14]. Notably, although a broad spectrum of MPs exists in the environment, current research on developmental toxicity remains predominantly focused on polystyrene microplastics (PS-MPs). This focus stems primarily from the comparative ease of PS particle synthesis, their high surface modifiability, and their established utility in laboratory models [15]. However, due to significant differences in size and surface characteristics, it is difficult to directly compare other MPs. Consequently, most of the mechanistic studies cited in this review are also dominated by PS-MPs, and systematic studies on other types of MPs need to be enhanced in the future.

## 2. Environmental Fate of Microplastics and Risks of Human Exposure During Developmental Stages

MP contamination is a growing environmental concern, with potential implications for ecosystems and human health [16]. MPs are widely distributed across atmospheric, terrestrial, and aquatic environments [17,18]—including the air we breathe [19]. They enter the human body through both direct and indirect exposure pathways, contributing to a range of adverse health effects [20]. Numerous studies have detected MPs in human feces [21], lungs, blood [22], placenta [13], and other tissues [23], as well as in breast milk, indicating widespread contamination of the human body.

### 2.1. Sources and Types of Microplastics

MPs are defined as synthetic polymer particles, films, and fibers with a diameter of <5 mm [10]. These particulates originate through two principal pathways: primary microplastics and secondary microplastics. Primary microplastics are manufactured due to industrial needs and are commonly found in personal care products, abrasive ingredients in cosmetics and cleaning products, as well as textiles and fiber garments [24]. Secondary microplastics are tiny plastic particles produced from large plastic waste that undergoes physical, chemical, and biological processes, causing splitting and fragmentation [25]. The morphological diversity of MPs is intrinsically linked to their emission sources and environmental weathering processes, including fragments, fibers, small particles, spheres, films, and foams [26]. MPs are predominantly composed of various synthetic polymers; common polymer types include polyethylene (PE), polypropylene (PP), polyvinyl chloride (PVC), polyethylene terephthalate (PET), polystyrene (PS), and polyamide (PA) [27,28]. Among them, polyethylene is the main type of MPs in the environment [29].

The annual global terrestrial microplastic emissions (number of particles) are 6.1 × 10^16^ particles/year (range 1.3 × 10^16^–1.1 × 10^17^), and for the ocean, emissions are 2.6 × 10^15^ particles/year (range 2.7 × 10^14^–5.0 × 10^15^) [30]. The total annual release of personal care products (PCPs) is about 12,000 tons, of which about 1500 tons enter water bodies through sewage treatment plants [31]. Household cleaning products (such as laundry detergents and toilet cleaners) release 3.88 × 10^12^ ± 1.35 × 10^12^ microplastic particles annually, with toilet cleaners contributing 56.44%, and about 63.40% being discharged directly into water bodies untreated [32].

### 2.2. Environmental Behavior

MPs are widely distributed in various environmental media such as water, soil, and the atmosphere, and their behavioral characteristics such as migration, adsorption, and degradation in the environment determine their potential environmental risks.

MPs migrate in response to physical, chemical, and biological factors. MPs have an isomerization effect, a phenomenon that is a major determinant of their transport, distribution, and bioavailability in the environment. The migration of MPs exacerbates the spread of pollution, and once in the ecosystem, MPs are resistant to traditional remediation strategies, resulting in their progressive environmental accumulation [33]. Owing to hydrophobicity and a high specific surface area, MPs possess a pronounced capacity to adsorb coexisting environmental pollutants [34]. They function not only in the form of a single pollutant in the environment, but also as vectors for complex pollutant mixtures, capable of simultaneous multi-pollutant sorption. For the same type of MPs, their adsorption capacity increases with decreasing particle size [35]. In addition, different types of MPs show distinct adsorption affinities for the same pollutant [36]. MPs undergo gradual degradation primarily via three pathways: biodegradation, photodegradation, and chemical degradation. Biodegradation is the key pathway, in which microorganisms decompose MPs polymers into small molecules by secreting enzymes, and eventually mineralize them into harmless substances such as carbon dioxide and water [37]. Distinct types of MPs have different degradation difficulties and speeds due to differences in their chemical structures. However, MPs degrade slowly in the environment and are not easily decomposed [38].

Environmental MPs enter the human body via multiple pathways, such as ingestion of contaminated water and food, inhalation, and trophic transfer. This exposure can interfere with normal physiological functions and inducing a series of health complications, encompassing organ-specific toxicities like neurotoxicity, hepatotoxicity, cardiotoxicity, and developmental reproductive toxicity [39]. Given the toxicity of MPs observed in environmental model organisms and their potential implications for human health, further investigation into their developmental toxicity is needed to better assess ecological and health risks.

### 2.3. Environmental Exposure Concentration and Exposure Risk Assessment of Microplastics

As consumers at the top of the food chain, humans consume more MPs than other organisms, primarily by breathing air and ingesting contaminated food containing MPs. Upon entering the body, MPs can cross biological barriers and accumulate in the digestive system and other organs. Researchers have discovered MPs in human sputum [40], placenta [41], blood [42,43,44], breast milk [45,46], liver and lungs, and other organ tissues [47,48,49,50,51,52] using various analytical techniques. A study on 17 human arterial samples (including coronary arteries) showed that the average concentration of MP was 118.66 ± 53.87 μ g/g tissue [22]. Using laser direct infrared spectroscopy (LD-IR) to analyze multiple types of human tissues, it was found that MPs ranging from 20 to 100 μm were commonly present in all detected tissues, with PVC being the most predominant polymer type and the highest abundance in lung tissue (average 14.19 ± 14.57 particles/gram) [53]. Among 17 placental samples, LD-IR analysis showed 100% detection of MP, with an average abundance of 2.70 ± 2.65 particles/gram (range 0.28–9.55 particles/gram), covering a total of 11 types of polymers [54]. Using micro-Raman spectroscopy technology, researchers have confirmed for the first time the presence of MP in human kidney tissue and urine samples, detecting particle sizes ranging from 1 to 29 μm and mainly composed of PE and PS. Open source software has been developed to assist in spectral comparison [55]. In another study on 50 placental samples, a total of 40 MP particles were detected in 31 samples, with an average particle size of 2.35 ± 1.25 µm [56]. Among the whole blood samples of 36 healthy adults, 88.9% were detected with MP (average 4.2 particles/mL), mainly consisting of PS and PP. High load (≥3 particles/mL) was associated with abnormal coagulation indicators and elevated inflammatory markers [57]. In addition, MP was first detected in menstrual blood and amniotic fluid [58], and was found in both testes and semen, with an average concentration of 11.60 ± 15.52 particles/g in testes and 0.23 ± 0.45 particles/mL in semen [59]. Based on comprehensive studies on human biomonitoring and exposure assessment, polyesters (especially PET), PE, PP, PA, and PVC are the most commonly detected polymers in microplastics ingested or inhaled by humans. In human samples (such as feces and gastrointestinal contents) and food chain-related studies, PET has been systematically reviewed as the most frequently detected polymer in the human body, followed by PA, polyurethane (PU), PP, and polyacrylate [60]. In atmospheric microplastic samples, PET and PVC are the most abundant types, followed by PP and PE [61]. Several studies have listed PE, PP, and PS as the main polymers for inhalation exposure [62].

**Figure 1:**
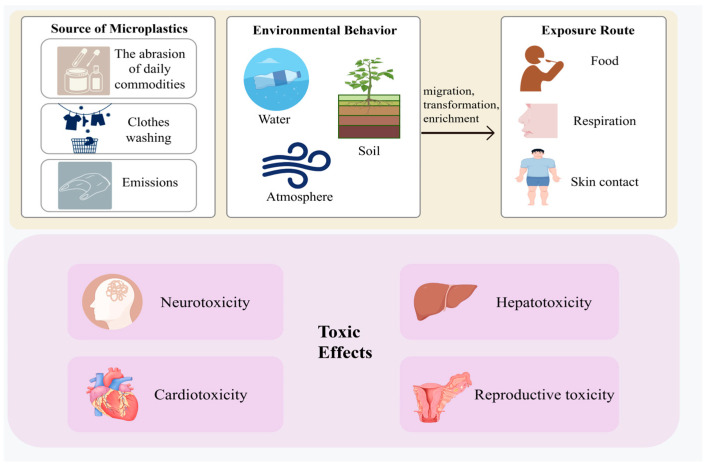
The environmental fate, human exposure, and developmental toxicity effects of MPs. The figure elements used were created using Figdraw (https://www.figdraw.com, copyright code: SRRITf40fc, accessed on 23 June 2026).

Studies have shown that MPs may pose a number of potential risks to human health: they may lead to problems such as inflammatory responses, oxidative stress, organ toxicity and apoptosis, and have been associated with a wide range of diseases such as cancer, cardiovascular disease, diabetes, autoimmune diseases, neurodegenerative diseases and reproductive disorders [63,64,65,66]. However, current research on human MPs still faces difficulties in pollution control, limitations in detection technology, doubts about quantitative reliability, and lack of standardization. Therefore, existing detection data should be interpreted cautiously as “preliminary evidence of exposure” rather than absolute quantitative conclusions. We should call for establishing standardized testing procedures, strengthening quality control measures, promoting the integration of multiple technologies for verification, and combining in vitro/animal experiments to explore the mechanisms of health effects in depth, providing direction for subsequent large-scale longitudinal research and risk assessment. Figure 1 systematically summarizes the environmental fate, human exposure, and developmental toxicity effects of MPs.

MPs could affect fertility in both males and females [67]. Owing to their small size and irregular shape, microplastic particles may cause direct tissue damage within the reproductive system and alter the microenvironment of reproductive organs. The reproductive toxicity induced by MPs has been shown to include reduced fertility [68], decreased sperm motility [69], declines in germ cell numbers, decreased offspring survival rates, and growth retardation [70]. In addition, microplastic particles can cause hypoxia in embryos by covering their surface; they can also accumulate in the yolk sac, interfering with nutrient uptake and thereby exerting adverse effects on embryonic development [41]; disrupt gamete cell membrane fluidity, hinder gamete binding, and cause abnormal growth and development and metabolic problems in offspring, resulting in significant reproductive toxicity to organisms at the three levels of gametes, embryos, and offspring [71,72]. Studies have shown that exposure to MPs exposure may be linked to various congenital disorders. For instance, exposure to MPs may disrupt the endocrine system, thereby affecting the reproductive development of the fetus [73]. Exposure may also perturb key embryonic signaling pathways, leading to structural anomalies such as congenital heart disease [74]. In zebrafish, early-life exposure to PS-MPs induces phenotypic alterations including shortened body length, reduced heart rate, and decreased spontaneous movement. Notably, amino acid-modified polystyrene microplastics (PS-NH_2_) showed higher developmental toxicity compared to conventional PS-MPs, suggesting that surface modification increases toxicity and causes abnormal larval development [75]. Surface modifications such as amination can markedly enhance the toxicity of MPs, suggesting that physicochemical characteristics including particle size, surface charge and additives are critical determinants of MP toxicity. These parameters therefore require refined consideration in subsequent risk assessment frameworks. In murine models, maternal exposure to PS during different stages of gestation and lactation was found to induce metabolic changes in the cortex and hippocampus, as well as reduce the number of deep-layer neurons and disrupt hippocampal synaptic structures in offspring [76]. Moreover, maternal exposure to PS-MPs at concentrations of 100 μg/L and 1000 μg/L and with particle sizes of 0.5 μm and 5 μm during pregnancy is associated with multifaceted alterations in offspring, including behavioral changes and abnormalities in physiological serum biomarkers, suggesting that such exposure may induce potential long-term developmental toxicity by interfering with key developmental processes. Notably, the toxic effects of the 5 μm PS-MPs are more pronounced than those of the 0.5 μm particles [72].

Transgenerational studies reveal that parental exposure to PS-MPs at different concentrations (1, 10, 100, 1000 beads/mL) and particle sizes (1.7, 6.8, 10.4, 19.0 μm) increases offspring larval mortality and delays development. After parental exposure to 1.7, 6.8, and 10.4 μm PS-MPs, the mortality rate of offspring larvae significantly increased, while 19.0 μm PS-MPs showed basically no significant intergenerational effects at any concentration, particularly when exposure occurs during the sensitive larval stage of parents, demonstrating the capacity of MPs to exert cross-generational effects [77]. In summary, current evidence indicates that MPs elicit developmental toxicity across diverse experimental models, resulting in growth retardation, morphological and structural abnormalities, and transgenerational effects. However, human evidence remains limited, and large-scale clinical studies are lacking. Future research priorities should include in-depth mechanistic analyses, refined environmental risk assessments, and rigorous evaluation of the potential impacts of MPs on human developmental health.

## 3. Developmental Toxicity of Microplastics Based on Stem Cell Modeling

Pluripotent stem cells (PSCs), including embryonic stem cells (ESCs) and induced pluripotent stem cells (iPSCs), offer a unique advantage in recapitulating early developmental processes in vitro and have become ideal models for developmental toxicity research [78]. Unlike traditional animal models, which struggle to accurately simulate human embryonic development due to species differences, PSC models are based on human cells. They avoid misjudgments of developmental pathways caused by species differences and directly simulate key events in human embryogenesis (such as lineage specialization and organ formation), thereby more reliably assessing the potential hazards of MPs to human development. As PSCs are increasingly used to predict the developmental toxicity of chemicals, they provide a platform for simulating early embryogenesis and overcoming the limitations of traditional animal studies [79]. MPs could accumulate in organisms and interfere with key biological processes, including stem cell self-renewal, proliferation, and differentiation, thereby disrupting normal tissue development and repair. Planarians contain abundant adult pluripotent stem cells, making them an ideal model for studying stem cell biology and regeneration. This model facilitates the establishment of a developmental toxicity screening system with stem cell function as the endpoint, providing a reference framework for assessing the potential risks of emerging pollutants such as MPs to the stem cell homeostasis of higher organisms, including humans. Exposure to PS-MPs mixed with liver homogenate significantly inhibited stem cell proliferation and differentiation in a planarian model [80].

The developmental toxicity of MPs is primarily manifested in stem cells through the disruption of critical processes such as proliferation and differentiation. Such interference may compromise stem cell self-renewal capacity, as well as their roles in tissue repair and regeneration.

### 3.1. Stem Cell Modeling: A Unique Biological Platform for Studying Developmental Toxicity

Stem cells could be systematically classified according to their origin and differentiation potential. ESCs are pluripotent stem cells derived from early embryos and capable of differentiating into a variety of cell types originating from the three germ layers (ectoderm, mesoderm, and endoderm). Induced pluripotent stem cells (iPSCs) are pluripotent stem cells obtained by reprogramming adult cells (e.g., somatic cells), which are functionally similar to embryonic stem cells and can be directed to differentiate into various types of functional cells. PSCs provide a key platform for studying early developmental mechanisms and disease modeling [81]. Adult stem cells (ASCs), also known as tissue-specific stem cells, are found in adult tissues and are responsible for tissue renewal and repair. They include mesenchymal stem cells (MSCs), hematopoietic stem cells (HSCs), neural stem cells (NSCs), and intestinal stem cells, etc., with different tissue-specific origins, which can be used to study tissue-specific development [82]. Organoid is a three-dimensional (3D) structure based on self-organized formation of stem cells, which can more realistically simulate the microstructure, cellular diversity, and function of an organ, and provides an advanced model for studying more complex developmental interactions and toxicity [83]. Studies have shown that ESCs can also be used to study the toxic effects of nanoparticles (e.g., silver nanoparticles) on early development [84]. iPSCs can also be used to model cardiovascular developmental toxicity and to study the effects of environmental pollutants on cardiac developmental processes [85]. Therefore, as a new type of environmental pollutant, the use of stem cell models to advance related research can directly assess the direct effects of MPs on the direction and efficiency of stem cell differentiation, as well as the function of differentiated cells, which can help to promote the development of microplastic toxicology.

### 3.2. Microplastics Disrupt Stem Cell Self-Renewal, Proliferation and Cellular Homeostasis

Exposure to MPs significantly impairs the proliferative capacity of human cells, as indicated by diminished cellular viability, reduced clonogenic potential, and disruption of cell cycle progression, and induces marked alterations in cellular morphology and compromises homeostasis [64,86]. Studies have shown that after exposing human alveolar A549 cells to PS-MPs results in a time-dependent decline in cell viability, a significant suppression of proliferation, and concomitant changes in cell morphology and structure [87]. Investigations utilizing charged PS on neural progenitor cells (NPCs) further reveal that treatment with 50 nm, positively charged particles (PS-NH_3_^+^) causes pronounced inhibition of proliferation, reflected in a lower proliferation rate. This effect is mechanistically linked to the induction of DNA damage, leading to a cell cycle arrest at the G1 phase [88]. Moreover, susceptibility to MP exposure varies among different stem cell types. Exposure of MSCs to PS-MPs induces marked reductions in cell viability, appears cellular senescence, and impairs proliferative capacity [89]. Furthermore, MPs possess the ability to infiltrate bone marrow and colonize the hematopoietic microenvironment. Such exposure can result in a decline in hematopoietic stem cell (HSC) numbers, elevated apoptosis, cell cycle dysregulation, disruption of proliferative homeostasis, and consequent damage to the hematopoietic system [90]. In vitro investigations using umbilical cord blood-derived HSCs demonstrate that PS, PE, PP and PVC compromise both self-renewal and clonogenic potential in a concentration-dependent manner, thereby adversely affecting hematopoietic function [91]. Nanoparticles potentially derived from the continuous environmental degradation of MPs [92]. PS-NPs were round under electron microscopy, with a primary size of 30 nm (agglomerated size of approximately 112–115 nm). Exposure of a human neural stem cell line (hNS1) to PS at concentrations of 0.5, 2.5, and 10 μg/mL triggers oxidative stress, leading to DNA damage, activation of apoptotic pathways, and ultimately neuronal impairment and associated functional alterations [93]. Evaluating the toxicity of PS-MPs via a human kidney organoid model reveals that PS-MPs exposure causes a significant reduction in organoid size, concomitant with decreased viability, diminished proliferation, and increased apoptosis [94]. In summary, exposure to MPs exerts pronounced anti-proliferative and homeostasis-disrupting effects across a broad spectrum of human cellular models, encompassing somatic cells, multiple adult stem cells, and organoid models. These findings suggest a potential threat posed by MPs to the physiological integrity of diverse human tissues and organs. A summary of this is presented in Table 1.

The physicochemical properties of MPs, including surface charge, hydrophobicity, and chemical additives, may independently or synergistically affect differentiation outcomes, but these factors still need to be explored. Particle size is a key determinant of the biological effects of MPs. Current research mainly focuses on micron sized (1–5 μm) or nanometer sized (<100 nm) particles, but lacks systematic comparisons of different size scales within the same stem cell model [95]. Nanoscale MPs (especially NPs produced by environmental degradation) may exhibit toxic characteristics different from micrometer sized particles due to their small size, high specific surface area, and strong transmembrane transport ability, including easier penetration of biological barriers, accumulation in tissues and organs, and induction of more pronounced oxidative stress and inflammatory responses [96]. However, the understanding of toxicity and mechanism differences between particle sizes is still limited at present. Future research should prioritize expanding the types and size ranges of MPs studied, using standardized experimental frameworks for systematic comparisons, and evaluating the comprehensive effects of MP mixtures related to the environment, in order to gain a more comprehensive understanding of the health risks associated with exposure to MPs.

### 3.3. Microplastics Inhibit Tissue-Specific Differentiation and Organogenesis

A central manifestation of the developmental toxicity of MPs is their interference with stem cell differentiation processes, hindering the maturation of stem cells toward specific lineages. Prolonged exposure to MPs facilitates their accumulation within human tissues and organs, potentially resulting in persistent dysfunction of stem cells and disruption of stem cell self-renewal and differentiation processes [97].

Cardiovascular and cardiac development: In human cell models, a 24 h co-culture of human induced pluripotent stem cells (hiPSCs) with polystyrene nanoplastics (PS, 40 nm and 200 nm; 1 × 10^9^ particles/mL) significantly impairs the development of ventricular valves derived from the differentiation of these cells, thereby indicating an elevated risk of cardiovascular pathologies [98]. Following cellular internalization, PS exerts cytotoxic effects on the cardiac differentiation of human embryonic stem cells (hESCs), perturbs the expression of developmentally associated genes, and consequently suppresses hESC differentiation into cardiomyocytes. This inhibition of cardiomyogenic differentiation and dysregulation of underlying regulatory mechanisms ultimately result in impaired differentiation efficiency and aberrant cellular morphology [99].

Skeletal and mesenchymal differentiation: Exposure of BMSCs to PS-MPs induces a dose-dependent decline in cell viability and a concurrent rise in cytotoxicity. This exposure also suppresses the osteogenic differentiation of BMSCs, evident as diminished bone formation coupled with enhanced osteoclast activity [100]. Exposure of PET-MPs of different sizes to human bone marrow mesenchymal stem cells (BMMSCs) and adipose mesenchymal stem cells (AMSCs) has been found to directly inhibit the growth capacity of the stem cells, as evidenced by an increase in apoptosis, a decrease in the rate of proliferation, and impaired directional differentiation into adipocytes, osteoblasts, and chondrocytes. However, the sensitivity of the two cell types was not compared in the study [101].

Neural development: In NSCs, exposure to various sizes of PS-MPs significantly reduces cellular activity by inhibiting proliferation. This toxicity further compromises differentiation potential, specifically reducing oligodendrocyte yield and impairing neuronal differentiation [102]. In addition, upon prolonged exposure to high concentrations of small-sized PS, NSCs exhibited marked cellular morphological alterations, concomitant with elevated cytotoxicity and cell death rates. Concurrently, the expression of proliferation marker genes was downregulated in a dose-dependent manner, while their capacity for differentiation into the neural lineage was slightly attenuated [103].

Hematopoietic development: Exposure of human CD34^+^ hematopoietic stem/progenitor cells (HSPCs) to PS-NPs demonstrated that PS-NPs undergo cellular internalization, induce decreased cell viability, and consequently compromise HSPC self-renewal, sustained proliferation, and multilineage differentiation potential [104].

Organoid models of organogenesis: Using a human iPSC-derived kidney organoid model, Zhang et al. (2025) [105] demonstrated that PS-MPs disrupted nephron formation and epithelial cell differentiation, with mitochondrial oxidative stress and activation of the Bcl-2/Bax/caspase-9/caspase-3 apoptotic pathway identified as key underlying mechanisms. The neurodevelopmental toxicity of MPs has been further substantiated by studies employing human iPSC-derived brain organoid models. Hua et al. (2022) [106] reported that long-term exposure to PS-MPs downregulated mature neuronal markers and cortical layer-specific genes (*TBR1*/*TBR2*) in cortical spheroids, providing early evidence for the adverse effects of MPs on human embryonic brain development. Extending these findings to other polymer types, Huang et al. (2025) [107] demonstrated that early-life exposure to polypropylene nanoplastics (PP-NPs) significantly inhibited neuronal differentiation and proliferation in human iPSC-derived cerebral organoids, with *CYSLTR1* and *PTH1R* identified as key molecular targets. Using a human iPSC-derived intestinal epithelial cell model, Brouwer et al. (2025) [108] systematically evaluated the toxicity of true-to-life MPs, including PET with titanium dioxide filler (PET-TiO_2_), PP with talc filler (PP-Talc), PVC, and polyamide (PA). Their results demonstrated that exposure to PP-Talc and PVC compromised intestinal epithelial barrier integrity, while PET-TiO_2_ and PA induced significant elevation of intracellular reactive oxygen species. Notably, the commonly used substitute PS and PLA did not exhibit detectable toxicity in this model, highlighting the critical importance of polymer type and filler composition in determining toxicological outcomes.

Beyond conventional plastics: In addition to conventional MPs, biodegradable alternatives such as polylactic acid (PLA) have also been shown to exert adverse effects on stem cell-related processes. Zhang et al. (2025) [109] reported that both PS-MPs and PLA-MPs impaired transzonal projections and oocyte maturation in murine models, with PLA-MPs exhibiting higher cytotoxicity in vitro, suggesting that biodegradable MPs are not necessarily safer than conventional ones.

In summary, existing evidence suggests that MPs, particularly PS-MPs, can impair cell survival, disrupt proliferation, and interfere with stem cell function in various human cell models. More importantly, MPs can disrupt the normal differentiation ability of different stem cell types in organs such as the cardiovascular, skeletal, neural, hematopoietic, and intestinal systems through various pathways (a summary of this is presented in Table 2), which is a key mechanistic factor for their developmental toxicity and long-term health hazards. However, the toxicity characteristics of different polymer types cannot be summarized solely from PS-based models [108]. In addition, new evidence regarding PLA suggests that biodegradable MPs themselves may not pose a lower risk than traditional MPs, and their environmental and health impacts deserve reassessment [109].

### 3.4. Mechanistic Study of Microplastic-Induced Developmental Toxicity Based on Stem Cells

A substantial body of research indicates that MPs exposure triggers a series of basal cytotoxic responses that converge on common mechanisms leading to functional impairment. Stem cell models have proven instrumental in dissecting these molecular pathways, revealing both conserved and polymer-specific mechanisms of developmental toxicity.

#### 3.4.1. Oxidative Stress and Mitochondrial Dysfunction

Oxidative stress represents a central mechanistic hub in MP-induced developmental toxicity. High dose short-term exposure of human embryonic stem cell (hESC)-derived cardiomyocytes (CMs) to PS-NPs revealed that accumulated PS-NPs adsorb amino acids onto their hydrophobic surfaces, induce protein misfolding, activate endoplasmic reticulum stress and oxidative stress, and jointly activate the apoptotic pathway. By interfering with RNA splicing and protein folding pathways, these PS-NPs disrupt calcium ion signals, ultimately leading to arrhythmia [110]. Similarly, low dose long-term exposure to human induced pluripotent stem cell-derived cardiomyocytes (hiPSC-CMs) to varying concentrations of polystyrene micro/nanoplastics (PS-MNPs) leads to a decrease in mitochondrial membrane potential, an increase in mitochondrial ROS, and the induction of oxidative stress, which in turn causes calcium handling disorders, myocardial contraction dysfunction, myocardial hypertrophy, and ultimately impaired cardiac function [111]. In three-dimensional cardiac organoids (COs) derived from human pluripotent stem cells (hPSCs), PS-MPs are taken up by cardiomyocytes, triggering oxidative stress and inflammatory reactions, leading to energy metabolism disorders, calcium homeostasis imbalance, increased cell death, and ultimately concentric myocardial hypertrophy and impaired heart function [112].

The mechanistic link between oxidative stress and developmental toxicity is further substantiated in kidney organoid models. Short-term exposure to PS-MPs disrupts the structural integrity of hiPSC-derived kidney organoids, induces mitochondrial oxidative stress, DNA damage, and Bcl-2/Bax/caspase-9/caspase-3 pathway-dependent apoptosis, leading to impaired nephron formation, abnormal epithelial differentiation, and structural abnormalities, thus posing potential risks to embryonic kidney development [105]. In liver organoids (LOs) differentiated from hPSCs, short-term low-dose exposure to PS-MPs can induce oxidative stress, mitochondrial dysfunction, and inflammation, leading to lipid metabolism disorders, hepatotoxicity, lipid accumulation, and steatosis, suggesting the possibility of further progression to liver fibrosis and liver cancer [113].

In summary, the particle size, exposure dose, exposure time, and model system of plastic particles all affect the toxicity performance and cause differences in the mechanism of action and toxic effects through oxidative stress.

#### 3.4.2. Apoptosis and Programmed Cell Death

Apoptosis emerges as a predominant mode of cell death across multiple stem cell and organoid models. In a three-dimensional kidney organoid model constructed from hPSCs, PS-MPs are internalized by cells, induce organoid volume reduction, and significantly upregulate *DDIT4*, which then inhibits the mTOR signaling pathway, activates autophagy and apoptosis, leading to developmental toxicity of renal organoids. However, the root causes of the differences in sensitivity were not explored in depth. This study provides preliminary molecular mechanistic evidence for the renal developmental toxicity caused by MPs [114]. Mechanistically, exposure to PS MPs, Bcl-2 expression decreases and Bax expression increases, leading to changes in mitochondrial membrane permeability, promoting the release of cytochrome c into the cytoplasm, thereby activating caspase-3 and inducing cell apoptosis [105]. In human endometrial organoids, MP exposure induces significant cardiomyocyte apoptosis and disrupts normal organoid growth patterns [115].

Beyond PS, other polymer types also trigger apoptotic pathways. PET-MPs exposure in human bone marrow mesenchymal stem cells (BMMSCs) and adipose mesenchymal stem cells (AMSCs) increases apoptosis rates while impairing directional differentiation into adipocytes, osteoblasts, and chondrocytes [101]. In the planarian model *Dugesia japonica*, PS-MPs exposure inhibits stem cell proliferation and differentiation, reduces mitotic stem cell proportions, and delays regenerative capacity, with apoptosis identified as a contributing mechanism [80].

In summary, apoptosis is the main mode of inducing cell death by MPs in various stem cell and organoid models, and it is also a common terminal pathway for MPs to produce toxicity across polymer types and different models (organoid, stem cell, regenerative models).

#### 3.4.3. Cell Cycle Arrest, Senescence, and Autophagy

Disruption of cell cycle progression and induction of senescence represent additional mechanistic layers. In NSCs, exposure to various sizes of PS-MPs significantly reduces cellular activity by inhibiting proliferation, with prolonged exposure to high concentrations of small-sized PS leading to elevated cytotoxicity, cell death, and dose-dependent downregulation of proliferation marker genes [102,103]. In BMSCs, PS-MPs induce cellular senescence accompanied by reduced proliferative capacity [100]. The DDIT4-mTOR-autophagy axis has been identified as a key pathway mediating MP-induced stem cell dysfunction, particularly in neural stem cells and kidney progenitor cells [97].

#### 3.4.4. DNA Damage and Genomic Instability

DNA damage serves as an upstream trigger for many of the aforementioned cellular responses. In neural progenitor cells (NPCs), exposure to 50 nm positively charged PS particles (PS-NH_3_^+^) induces DNA damage, leading to G1 phase cell cycle arrest and pronounced inhibition of proliferation [88]. In hematopoietic stem/progenitor cells (HSPCs), PS-NPs undergo cellular internalization, induce decreased cell viability, and compromise self-renewal, sustained proliferation, and multilineage differentiation potential, with DNA damage implicated as a contributing mechanism [104]. The transgenerational reproductive toxicity induced by PLA-MPs in *C. elegans* involves dysregulation of DNA damage-related genes (*cep-1*, *hus-1*, *mrt-2*, *clk-2*) alongside apoptosis-related genes, suggesting that genomic instability may mediate heritable effects [116].

#### 3.4.5. Receptor-Mediated Signaling and Pathway-Specific Mechanisms

Emerging evidence reveals that MPs can exert toxicity through specific receptor-mediated mechanisms. Huang et al. (2025) [107] demonstrated that polypropylene nanoplastics (PP-NPs) bind to *CYSLTR1* (cysteinyl leukotriene receptor 1) and *PTH1R* (parathyroid hormone 1 receptor) in human iPSC-derived cerebral organoids, disrupting the neuroactive ligand-receptor interaction pathway and impairing neuronal differentiation and proliferation. This represents the first identification of specific molecular targets for non-PS nanoplastics in neurodevelopmental toxicity.

The gut microbiota-metabolite-HSC axis represents a novel indirect mechanism of stem cell toxicity. Jiang et al. (2024) [90] demonstrated that long-term microplastic exposure disrupts gut microbiota composition, reducing Rikenellaceae abundance and hypoxanthine levels, which subsequently inactivates the *HPRT-Wnt* signaling axis in bone marrow HSCs, impairing self-renewal and reconstitution capacity. This mechanism was validated through rescue experiments with rikenellaceae or hypoxanthine supplementation, establishing a causal gut microbiota-stem cell axis.

#### 3.4.6. Polymer-Specific and Weathering-Dependent Mechanisms

The role of environmental weathering in modulating toxicity is exemplified by Manabe et al. (2025) [117], who showed that surface-degraded PE and PVC micro/nanoplastics induce ferroptosis-related gene expression, increase ROS levels, and elevate lipid peroxidation, while pristine particles of the same polymers exhibit no such effects. This highlights that aged particles may possess distinct and enhanced toxicity profiles compared to pristine model particles.

#### 3.4.7. Protein Corona and Additive Effects

The formation of a protein corona on microplastic surfaces critically modulates cellular responses. Brouwer et al. (2025) [108] demonstrated that the protein corona composition on true-to-life MPs (PET-TiO_2_, PP-Talc, PVC, PA) correlates strongly with biological outcomes. PP-Talc surfaces were enriched with pro-inflammatory proteins, correlating with IL-6 secretion up to 8 times that of controls; PVC bound occludin, a tight junction protein, potentially explaining its barrier-disrupting effects. This work establishes protein corona fingerprinting as a predictive tool for hazard ranking of different polymer types.

In summary, stem cell and organoid models have revealed a complex landscape of mechanisms underlying MP-induced developmental toxicity. These mechanisms span multiple levels of biological organization—from molecular initiating events to cellular responses to tissue-level outcomes. Nevertheless, systematic comparisons across different polymer types and size ranges within the same stem cell model remain scarce. Although PSC models show promise as alternatives to animal testing for developmental toxicity evaluation, their in vitro systems still have limited physiological relevance. Most existing studies primarily focus on acute exposure outcomes, whereas systematic evidence remains scarce regarding the cumulative effects of environmental long-term low-dose exposure, intergenerational reproductive toxicity, and the specific damage mechanisms underlying reproductive organ injury.

In addition, the application of stem cell models in the field of developmental toxicology is still in the exploration and optimization stage. There are still several key limitations in the application of organoid systems in developmental toxicology: incomplete maturation of organ structure and function [118]; limited integration ability of immune cells; lack of functional vascular networks; difficulty in constructing long-term/chronic low-dose models that meet real exposure scenarios; significant inter-batch variability affecting experimental reproducibility; and difficulty in effectively simulating complex maternal–fetal interfaces. Although organoids have shown better physiological correlations than traditional two-dimensional culture and animal models in simulating specific organ microenvironments, cell heterogeneity, and donor-specific responses, they still belong to simplified in vitro approximation models overall. Due to the lack of standardized protocols, insufficient validation of predictive capabilities, and incomplete simulation of cross-organ interactions, organoid systems have not yet passed regulatory validation and cannot replace fully validated developmental toxicology testing methods [119].

Future research should prioritize: (1) use of standardized stem cell and organoid platforms to enable direct cross-polymer comparisons; (2) incorporation of environmentally relevant MPs, including aged particles and additive-containing formulations; (3) elucidation of the molecular pathways underlying polymer-specific and weathering-dependent toxicities; (4) integration of mechanisms into Adverse Outcome Pathway (AOP) frameworks for regulatory application; (5) deep analysis of toxicity mechanism; (6) strengthen the physiological maturity and validation of the model. Addressing these gaps will be essential for translating mechanistic findings into robust human health risk assessments. A summary of mechanistic findings is presented in Table 3.

## 4. Application of Modern Toxicological Analytical Methods to Microplastics

To systematically assess the biotoxicity of MPs, in vitro cellular models serve as an alternative or complementary tool to in vivo experiments. An in vitro modeling study based on two skin cell lines confirmed the dermal toxicity of NPs, which can damage skin cells, penetrate and compromise the skin barrier, and initiate inflammatory responses, thereby providing novel evidence for the dermal toxicity of MPs [120]. Studies based on the 3D blood–brain barrier (BBB) model revealed that PS-MPs exhibited more pronounced barrier penetration and disruptive effects under TNF-α stimulation-an observation that could not be captured by the 2D model. In addition, the size-dependent toxicity of PS-MPs also differed significantly between the 2D and 3D models, highlighting the necessity of in vitro modeling for the accurate assessment of MP-related environmental pollutant toxicity [121].

However, modeling can only reveal toxic phenomena to a certain extent; to elucidate the underlying intrinsic molecular mechanisms, highly efficient histological techniques are required. By integrating multi-omics technologies such as transcriptomics, metabolomics, proteomics, and microbiomics, a systematic analysis of the molecular interference effects induced by microplastic exposure on the developmental processes of organisms can be achieved. Metabolomic studies have shown that when *Crassostrea gigas* is exposed to PS-MPs, its metabolic profile is significantly regulated by the concentration of MPs, and such exposure interferes with the core pathways of amino acid, lipid, and glucose metabolism in *C. gigas* [122]. Ultra-high performance liquid chromatography-tandem mass spectrometry (UHPLC-MS/MS)-based metabolomic analysis demonstrated that exposure of zebrafish to PS-MPs disrupts the homeostasis of amino acid, carbohydrate, and nucleotide metabolism in the intestinal tissue [123]. Lipidomic analysis of mice exposed to PS-MPs indicated that the levels of hepatic free fatty acids, partial neutral lipids, glycerophospholipids, and sphingolipids were significantly elevated in the exposed group, which was accompanied by impaired glucose tolerance. Transcriptomic analysis further illustrated that the altered hepatic gene expression profiles were significantly enriched in lipid metabolism and endoplasmic reticulum stress pathways, and the differentially expressed genes exhibited a close correlation with the observed lipid metabolic alterations [124]. Joint microbiome-metabolome analyses demonstrated that exposure to polylactic acid microplastics (PLA-MPs) can indirectly disrupt the host’s systemic metabolic homeostasis by altering the intestinal flora structure of mice, ultimately impairing their developmental processes [125]. After exposing LOs derived from human embryonic stem cells to polypropylene microplastics (PP-MPs), combined transcriptomic and metabolomic techniques were used to analyze changes in gene expression and metabolites in LOs and in vivo samples. The results revealed that low-dose PP-MPs, especially amorphous polypropylene microplastics (aPP), interfere with hepatic homocysteine metabolism—a metabolic process that plays a key role in inducing mitochondrial dysfunction and metabolic disorders. However, independent and repeated evidence was not cited in the study [126].

At the cutting edge of the ongoing advancement of the aforementioned technologies, organ chips systems exhibit the capability to recapitulate the microenvironmental characteristics of human organs. This unique feature enables the dynamic monitoring of toxicological responses induced by MPs at the organ level, thereby providing developmental toxicity data that more closely approximates the physiological reality of the human body [127]. A series of studies leveraging an integrated microfluidic lung-on-a-chip system have facilitated gradient-modulated investigations into the dynamic toxicological profiles of NPs in the pulmonary system. It was observed that exposure to PS-NPs at a high concentration (>50 µg/mL) elicited pronounced nanotoxic effects in alveolar epithelial cells, whereas low-dose exposure triggered the activation of cellular defense mechanisms. This model not only establishes a platform for organ-level toxicological evaluation but also sheds light on the potential health risks posed by NPs to the human body [128].

**Figure 2:**
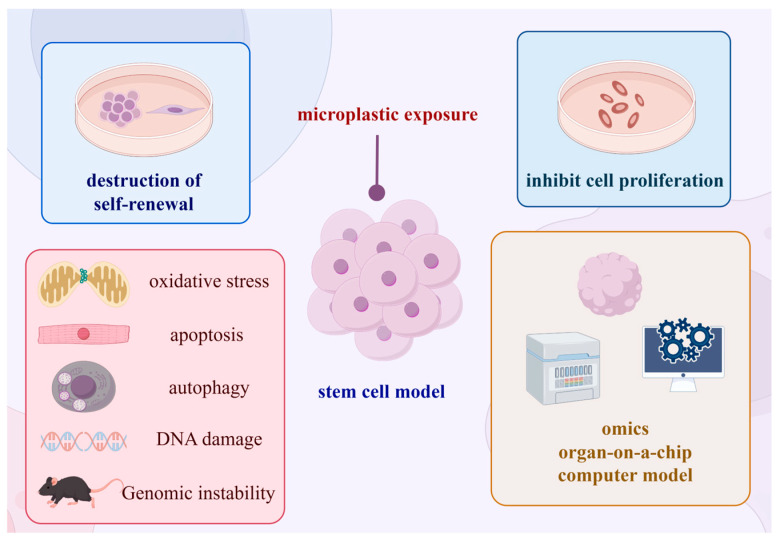
The mechanism of developmental toxicity induced by microplastics based on stem cells and the application of modern toxicological analysis methods in this field. The figure elements used was permitted by Figdraw (https://www.figdraw.com, copyright code: UASTI9aa29, accessed on 23 June 2026).

To further integrate multi-dimensional data and predict molecular interactions, computer models exhibit robust capabilities in data integration and simulation. Specifically, computational toxicology models serve to predict developmental toxicity while minimizing the reliance on animal experiments [129]. Molecular docking and molecular dynamics (MD) modeling are widely used to study the interaction of MPs and their additives with biomolecules. A toxicity study targeting 10 common polymeric MPs—such as PS, PP, and PVC—adopted a combined strategy of molecular docking, MD modeling, and fractional factorial design. This integrated method enabled a systematic evaluation of the hazardous effects of the aforementioned MPs, and the results indicated that polycarbonate (PC) exhibited the highest toxicity to zebrafish [130]. In experiments involving the exposure of zebrafish to amino-modified polystyrene microplastics (PS-NH_2_), molecular docking assays verified the presence of high binding energy between PS-NH_2_ and the anti-apoptotic Bcl-2 protein. This critical molecular interaction finding serves as a fundamental basis for elucidating the toxicological mechanism of PS-NH_2_ during the early developmental stages of zebrafish [75]. To elucidate the combinatorial toxic effects of PS-MPs and perfluorooctanoic acid (PFOA) in zebrafish, an integrated analytical approach employing transcriptomics and molecular dynamics simulations was implemented. Transcriptomic analysis demonstrated that PS-MPs not only exacerbated PFOA-induced inhibition of neurotransmitter release in a particle size-dependent manner but also significantly suppressed amino acid metabolic pathways and detoxification-related signaling cascades. Furthermore, MD simulations further elucidated that PS-MPs regulated the bioaccumulation characteristics and toxicity profiles of PFOA by increasing the binding energy and altering the conformational flexibility of target proteins [131]. Linear modeling of larval development in *Chironomus riparius* following exposure to PE-MPs (size < 63 μm, concentration 2.5 g/kg) demonstrated that MP toxicity was enhanced under combined temperature and nutritional stress, specifically at low temperatures or under food deprivation conditions [132]. Separately, simulations employing toxicokinetic-toxicodynamic (TK/TD) modeling revealed that the in vivo accumulation kinetics of PS-MPs in mice and their consequent multiorgan toxicity were strongly influenced by particle size, identifying size as a critical determinant of their TK/TD profiles [133]. For the purpose of evaluating the environmental risks posed by MPs, several studies have predicted the size-dependent bioaccumulation trends of MPs through the development of kinetic models constructing MP accumulation and elimination in fly larvae, coupled with the integration of particle distribution parameters [134]. Figure 2 illustrates the developmental toxicity mechanism induced by stem cell-based microplastics and the application of modern toxicology analysis methods in this field.

It is noteworthy that existing in vitro cell models (including conventional two-dimensional cultures) and partial three-dimensional models still lack adequate simulation capacity for the combined exposure scenarios, long-term low-dose cumulative toxic effects, and trans-barrier transport behaviors of MPs as well as their associated polymer additives such as bisphenols. In addition, in-depth mechanistic research integrating multi-omics analysis and functional validation remains scarce.

Although organ-on-a-chip technology has overcome the inherent drawbacks of conventional in vitro models and better recapitulates the human physiological microenvironment, it still confronts several bottlenecks, including inadequate fabrication standardization, imperfect multi-organ integrated systems, and restricted capacity for long-term dynamic exposure simulation. At present, this technology has not yet been applied to large-scale and standardized high-throughput toxicological assessment, rendering it difficult to fully and accurately reproduce the actual toxic outcomes and systemic health risks induced by multi-pathway MPs exposure in humans.

## 5. Summary and Outlook

This review systematically delineates the profound implications of MPs for the developmental health of diverse species. Integrated findings demonstrate that MPs, following bioaccumulation, confer multisystemic developmental risks through mechanisms that include disrupting stem cell functions (e.g., inhibition of self-renewal and differentiation), inducing oxidative stress, inflammation, and other mechanisms. Stem cell-based modeling and state-of-the-art toxicological methodologies have served as pivotal tools in elucidating these mechanistic underpinnings.

Synthetic MPs are extensively dispersed throughout aquatic and terrestrial ecosystems, leading to developmental inhibition, reduced reproductive capacity, immune dysfunction, and intestinal barrier impairment. PLA, recognized as a “biodegradable” plastic, also presents considerable ecological risks through its micro- and nanoparticles. In comparison to MPs, NPs (<100 nm) demonstrate enhanced biological penetration, transgenerational toxicity, and molecular interference capabilities owing to their smaller size and greater specific surface area. Environmentally derived NPs can significantly disrupt the detoxification and immune gene expression in bivalves even at low concentrations, exhibiting a more pronounced effect than MPs. The toxicity response of artificially synthesized NPs differs from that of naturally occurring particles in the environment, underscoring the necessity of employing environmentally relevant particles and concentrations for risk assessment. Furthermore, the combined exposure to micro- and NPs often yields synergistic effects, while surface modifications further exacerbate the neural and intestinal toxicity of NPs. In conclusion, the ecological and health risks associated with plastic particles are highly contingent upon the polymer type, size, aging state, surface properties, and co-exposure conditions.

However, this review also highlights a significant limitation of current research: the current literature tends to focus on PS-MPs, mainly due to their well-defined physicochemical properties and commercial availability. Although emerging research on other polymer types such as PET, PE, PP, and PVC has begun to reveal similar toxic effects, there is still a lack of systematic comparative studies. A more fundamental problem lies in the systematic methodological limitations of current MPs toxicology research. On the one hand, laboratory exposure experiments often use extremely high concentrations (e.g., 10–500 mg/L), far exceeding actual environmental levels, while actual human exposure data show that the average quantifiable concentration of MPs in blood is 1.6 μg/mL [42], and the average concentration in bone marrow is 51.29 μg/g [135], with the average concentrations of MPs in indoor PM_2.5_ and PM_10_ being 0.51 μg/m^3^ and 1.14 μg/m^3^, respectively [136]. It is worth noting that human liver organoid studies have attempted to use 0.25 μg/mL PS-MPs exposure, which is equivalent to environmental exposure levels, and have observed lipid metabolism disorders and inflammatory responses [113]. PP and aPP can interfere with mitochondrial function and homocysteine metabolism at 75 ng/mL (close to the endogenous exposure level in the human body) [126]. In contrast, the concentration used in conventional stem cell experiments (such as 250 μg/mL) is about 156 times higher than the measured mean in blood (1.6 μg/mL), which deviates from real exposure scenarios and limits the reliability of extrapolation of in vitro toxicity results to population health risks. On the other hand, research heavily relies on commercially available monodisperse spherical PS particles, whereas MPs in real environments exhibit high heterogeneity, including polymer diversity, irregular morphology, and natural weathering characteristics, which cannot simulate the “non-uniform” system that the human body is actually exposed to. This simplified model has limited applicability under real exposure conditions, weakening the extrapolative power of research conclusions to human health risk assessment. Therefore, future research should expand its scope to cover a wider range of MP types in order to comprehensively evaluate their developmental toxicity.

Furthermore, notable knowledge gaps persist in the current body of research, specifically encompassing three core aspects: (1) toxicokinetic behavior of MP–chemical co-pollutant complexes; (2) the transgenerational epigenetic mechanisms underlying MP-induced toxicity; (3) the biological effects of long-term exposure to low-dose MPs. In future toxicology research, stem cell-derived organoids and organ-on-a-chip models should be integrated with multi-omics and computational biology approaches to systematically develop and validate quantitative adverse outcome pathways (qAOPs). This type of humanized model provides high-fidelity mechanistic data for AOP frameworks by accurately simulating the physiological microenvironment, cell heterogeneity, and individual-specific responses, significantly enhancing the analytical ability of toxicity pathways. Taking the assessment of developmental neurotoxicity (DNT) as an example, cortical organoids (COs) and neural stem cell organoids (NSCOs) derived from hiPSCs have been used to identify key events of thyroid hormone-interfering chemicals (such as neuronal precursor cell proliferation inhibition and synaptic formation disorders), providing direct mechanistic evidence for non-animal substitution strategies in the OECD testing guidelines. Under the Integrated Approaches to Testing and Assessment (IATA) framework, organ chips can serve as the core testing module, integrating real-time sensing data, organoid responses, and computational predictions to achieve multi-evidence-weighted toxicity assessment, improving decision-making efficiency and scientific rigor. This type of integrated data can also provide an empirical basis for broader updates of OECD testing guidelines—for example, muscle organoid and joint chip models have been used to evaluate the feasibility of alternative methods recognized by regulatory agencies, accelerating the standardization process of non-animal testing strategies. As a key pillar of non-animal testing systems, this technology not only reduces reliance on traditional animal experiments, but also supports the implementation of next-generation risk assessment (NGRA) frameworks by generating reproducible and comparable human physiological data. It should be emphasized that with scientific progress, public communication needs to rely on solid scientific evidence (such as organotoxicity data and real-world environmental exposure results) to enhance the accuracy of risk awareness and strengthen the scientific foundation of evidence-based environmental policies and sustainable governance.

## Figures and Tables

**Table 1:** Impact of microplastics on self-renewal of stem cells.

Cell	Types of Microplastics	Particle Size	Exposure Concentrations	Toxic Effects	References
MSCs	PS	1 μm	0.1, 0.5, 1.0 mg/mL	Cell viability decreases and cellular senescence occurs	[89]
HSCs	PS, PMMA, PE	500 nm	0.01 mg/100 μL, 0.1 mg/100 μL	Increased apoptosis, cell cycle abnormalities, hematopoietic system damage	[90]
hNS1	PS	30 nm	0.5, 2.5, 10 μg/mL	DNA damage, apoptosis activation	[93]
human iPSC-derived kidney organoids	PS	1 μm	0, 0.625, 1.25, 2.5, 5, 10, 20 μg/mL	There was a significant reduction in organoid size, decreased cell viability, diminished proliferative capacity, and an increase in apoptosis	[94]

**Table 2:** Impact of microplastics on tissue-specific differentiation.

Organ	Cell	Types of Microplastics	Particle Size	Exposure Concentrations	Toxic Effects	References
Heart	hiPSCs	PS	40 nm, 200 nm	1 × 10^9^ particles/mL	Damage to ventricular valve development	[98]
Heart	hESCs	PS	28.65 ± 0.51 nm	5, 20 μg/mL	Inhibiting differentiation towards cardiomyocytes leads to reduced differentiation efficiency and abnormal cell morphology	[99]
Skeleton	BMSCs	PS	5 µm	30, 60 µg/mL	Inhibit osteogenic differentiation and enhance osteoclast activity	[100]
Skeleton	BMMSCs, AMSCs	PET	<1 μm (PET1), <2.6 μm (PET2.6)	10 μg/mL	Increased cell apoptosis, reduced proliferation rate, and impaired directed differentiation into adipocytes, osteoblasts, and chondrocytes	[101]
Nerve	NSCs	PS	0.1, 1, 2 μm	20, 40, 100 μg/mL	Reduced cell viability and impaired neuronal differentiation	[102]
Nerve	NSCs	PS	30 nm	0.5, 2.5, 10 μg/mL	The cell morphology changes, the proliferation ability decreases, and the differentiation towards the neural lineage weakens	[103]
Hemopoietic system	HSPCs	PS	80 nm	0.1 mg/mL	Damage to self-renewal, sustained proliferation, and multipotential differentiation potential	[104]
Kidney	human iPSC-derived kidney organoids	PS	1 μm	0, 1.25, 2.5, 5, 10, 20 μg/mL	Disrupting the formation of renal tubules and the differentiation of epithelial cells	[105]
Brain	human iPSC-derived forebrain organoids	PS	1, 10 µm	5, 50, 100 µg/mL	The cell survival rate decreases	[106]
Brain	human iPSC-derived cerebral organoids	PP	100 nm	0, 10, 25, 50 µg/mL	Inhibit the differentiation and proliferation of neurons in brain organoids	[107]
Intestine	human iPSC-derived intestinal model	PET-TiO_2_, PP-Talc, PVC, PA, PS, PLA	PS: 1152 ± 120 nm PP-Talc: 717 ± 292 nm PLA: 317 ± 27 nm PVC: 976 ± 13 nm PET-TiO_2_: 418 ± 37 nm PA: 477 ± 34 nm	125 μg/mL	PP-Talc and PVC can damage the integrity of the intestinal epithelial barrier, while PET-TiO_2_ and PA induce a significant increase in intracellular reactive oxygen species	[108]

**Table 3:** Impact of microplastics on cytotoxic mechanisms.

Mechanism	Cell	Types of Microplastics	Particle Size	Exposure Concentrations	Toxic Effects	References
**Oxidative stress and mitochondrial dysfunction**	human ESC-derived cardiomyocytes	PS	50 nm, 500 nm	10, 20, 40 μg/mL	arrhythmia	[110]
	human iPSC-derived cardiomyocytes	PS	0.05, 1 μm	0, 0.1, 1,10, 1000 μg/L	Contraction dysfunction, myocardial hypertrophy	[111]
	human PSC-derived cardiac organoids	PS	1 μm	0.025, 0.25, 2.5 µg/mL	Concentric myocardial hypertrophy	[112]
	human PSC-derived liver organoids	PS	1 μm	0.25, 2.5, 25 μg/mL	Hepatic steatosis, indicating liver fibrosis	[113]
	human iPSC-derived kidney organoids	PS	1 μm	0, 1.25, 2.5, 5, 10, 20 μg/mL	Abnormal renal development	[105]
**Apoptosis and programmed cell death**	human iPSC-derived kidney organoids	PS	1 μm	1.25–10 μg/mL	DDIT4/mTOR axis simultaneously drives autophagy and apoptosis	[114]
	human iPSC-derived kidney organoids	PS	1 μm	0, 1.25, 2.5, 5, 10, 20 μg/mL	Oxidative stress-induced Bcl-2/Bax/caspase pathway specifically mediates apoptosis	[105]
	human endometrial organoids	PS	2 μm	5, 50 μg/mL	Endometritis and activation of cell apoptosis	[115]
	BMMSCs, AMSCs	PET	<1 μm (PET1), <2.6 μm (PET2.6)	10 μg/mL	Changes in differentiation potential, cellular aging, and apoptosis	[101]
	*Dugesia japonica*	PS	10, 1, 0.1 μm	10, 50, 100 μg/mg of liver homogenate to prepare food mixtures	Inhibit the proliferation and differentiation of stem cells	[80]
**Cell cycle arrest, senescence, and autophagy**	NSCs	PS	0.1, 1, 2 μm	20, 40, 100 μg/mL	Cell membrane damage, cell cycle arrest	[102]
	NSCs	PS	30 nm	0.5, 2.5, 10 μg/mL	Apoptosis and reduced cell proliferation	[103]
	BMSCs	PS	5 µm	30, 60 µg/mL	Induce cell senescence and reduce proliferation capacity	[100]
**DNA damage and genomic instability**	NPCs	PS	50 nm	100, 200, 500 μg/mL	Inducing DNA damage, leading to cell cycle arrest	[88]
	HSPCs	PS	80 nm	0.1 mg/mL	Cell viability decreases, and DNA methylation changes	[104]
	*Caenorhabditis elegans*	PLA	2.92 ± 0.56 μm	1, 10, 100 μg/L	Inducing transgenerational reproductive toxicity, and transgenerational activation of DNA-damaging genes	[116]
**Receptor-mediated signaling and pathway-specific mechanisms**	human iPSC-derived cerebral organoids	PP	100 nm	0, 10, 25, 50 µg/mL	Disrupting the neuroactive ligand-receptor interaction pathway impairs the differentiation and proliferation of neurons	[107]
	HSCs	PS, PMMA, PE	500 nm,	100 μg/ mL, 250 μg/mL	Downregulation of Wnt pathway-related gene expression	[90]
**Polymer-specific and weathering-dependent mechanisms**	Mouse macrophage-like cell line RAW264.7	PE, PVC	PE/PVC MP: 100–230 μm; PE/PVC NP: 300–600 nm	MP: 10–25 mg/mL NP: 1.25–2.5 mg/mL	Both PE and PVC exhibit enhanced toxicity after degradation, promoting the accumulation of peroxides and lipids	[117]
**Protein corona and additive effects**	human iPSC-derived intestinal model	PET-TiO_2_, PP-Talc, PVC, PA, PS, PLA	-	125 μg/mL	The increase in the secretion of IL-6 and IL-8 is associated with pro-inflammatory proteins in the protein corona of microplastics	[108]

## Data Availability

No new data were created or analyzed in this study. Data sharing is not applicable to this article.

## References

[1] Kajal S., Thakur S. (2024). Coexistence of microplastics and heavy metals in soil: Occurrence, transport, key interactions and effect on plants. Environ. Res..

[2] Smriti, Kushwaha J.P., Singh N. (2025). Electrochemical remediation of microplastics: Progress and prospects in water treatment. J. Contam. Hydrol..

[3] Li Y., Tao L., Wang Q., Wang F., Li G., Song M. (2023). Potential Health Impact of Microplastics: A Review of Environmental Distribution, Human Exposure, and Toxic Effects. Environ. Health.

[4] Akdogan Z., Guven B. (2019). Microplastics in the environment: A critical review of current understanding and identification of future research needs. Environ. Pollut..

[5] Thacharodi A., Hassan S., Meenatchi R., Bhat M.A., Hussain N., Arockiaraj J., Ngo H.H., Sharma A., Nguyen H.T., Pugazhendhi A. (2024). Mitigating microplastic pollution: A critical review on the effects, remediation, and utilization strategies of microplastics. J. Environ. Manag..

[6] Kalčíková G., Bundschuh M. (2022). Aquatic Biofilms—Sink or Source of Microplastics? A Critical Reflection on Current Knowledge. Environ. Toxicol. Chem..

[7] Tang K.H.D., Li R. (2024). Aged Microplastics and Antibiotic Resistance Genes: A Review of Aging Effects on Their Interactions. Antibiotics.

[8] Zhang Z., Meng J., Tian J., Li N., Chen Z., Yun X., Song D., Li F., Duan S., Zhang L. (2024). Reproductive and developmental implications of micro- and nanoplastic internalization: Recent advances and perspectives. Ecotoxicol. Environ. Saf..

[9] Yu F., Wu J., Wang H., Bao Y., Xing H., Ye W., Li X., Huang M. (2024). Interaction of microplastics with perfluoroalkyl and polyfluoroalkyl substances in water: A review of the fate, mechanisms and toxicity. Sci. Total Environ..

[10] Wang C., Zhao J., Xing B. (2021). Environmental source, fate, and toxicity of microplastics. J. Hazard. Mater..

[11] Rendell-Bhatti F., Paganos P., Pouch A., Mitchell C., D’Aniello S., Godley B.J., Pazdro K., Arnone M.I., Jimenez-Guri E. (2021). Developmental toxicity of plastic leachates on the sea urchin *Paracentrotus lividus*. Environ. Pollut..

[12] Ding P., Xiang C., Yao Q., Li X., Zhang J., Yin R., Zhang L., Li A.J., Hu G. (2024). Aged polystyrene microplastics exposure affects apoptosis via inducing mitochondrial dysfunction and oxidative stress in early life of zebrafish. J. Environ. Manag..

[13] Luo Q., Tan H., Ye M., Jho E.H., Wang P., Iqbal B., Zhao X., Shi H., Lu H., Li G. (2025). Microplastics as an emerging threat to human health: An overview of potential health impacts. J. Environ. Manag..

[14] Zhang B., Chen L., Chao J., Yang X., Wang Q. (2020). Research Progress of Microplastics in Freshwater Sediments in China. Environ. Sci. Pollut. Res. Int..

[15] Chen H., Chen X., Gu Y., Jiang Y., Guo H., Chen J., Yu J., Wang C., Chen C., Li H. (2024). Transgenerational reproductive toxicity induced by carboxyl and amino charged microplastics at environmental concentrations in *Caenorhabditis elegans*: Involvement of histone methylation. Sci. Total Environ..

[16] Paul I., Mondal P., Haldar D., Halder G. (2024). Beyond the cradle-Amidst microplastics and the ongoing peril during pregnancy and neonatal stages: A holistic review. J. Hazard. Mater..

[17] Chanda M., Bathi J.R., Khan E., Katyal D., Danquah M. (2024). Microplastics in ecosystems: Critical review of occurrence, distribution, toxicity, fate, transport, and advances in experimental and computational studies in surface and subsurface water. J. Environ. Manag..

[18] Liu Y., Nie Z., Meng Y., Liu G., Chen Y., Chai G. (2025). Influence of meteorological conditions on atmospheric microplastic transport and deposition. Environ. Res..

[19] Sharaf Din K., Khokhar M.F., Butt S.I., Qadir A., Younas F. (2024). Exploration of microplastic concentration in indoor and outdoor air samples: Morphological, polymeric, and elemental analysis. Sci. Total Environ..

[20] Lee Y.H., Zheng C.M., Wang Y.J., Wang Y.L., Chiu H.W. (2025). Effects of microplastics and nanoplastics on the kidney and cardiovascular system. Nat. Rev. Nephrol..

[21] Schwabl P., Köppel S., Königshofer P., Bucsics T., Trauner M., Reiberger T., Liebmann B. (2019). Detection of Various Microplastics in Human Stool: A Prospective Case Series. Ann. Intern. Med..

[22] Liu S., Wang C., Yang Y., Du Z., Li L., Zhang M., Ni S., Yue Z., Yang K., Wang Y. (2024). Microplastics in three types of human arteries detected by pyrolysis-gas chromatography/mass spectrometry (Py-GC/MS). J. Hazard. Mater..

[23] Yang Q., Peng Y., Wu X., Cao X., Zhang P., Liang Z., Zhang J., Zhang Y., Gao P., Fu Y. (2025). Microplastics in human skeletal tissues: Presence, distribution and health implications. Environ. Int..

[24] Deng X., Gui Y., Zhao L. (2025). The micro(nano)plastics perspective: Exploring cancer development and therapy. Mol. Cancer.

[25] da Silva V.H., Murphy F., Amigo J.M., Stedmon C., Strand J. (2020). Classification and Quantification of Microplastics (<100 μm) Using a Focal Plane Array-Fourier Transform Infrared Imaging System and Machine Learning. Anal. Chem..

[26] Godoy V., Martín-Lara M.A., Calero M., Blázquez G. (2019). Physical-chemical characterization of microplastics present in some exfoliating products from Spain. Mar. Pollut. Bull..

[27] Liu Z., Liu X., Bai Y., Wei H., Lu J. (2023). Spatiotemporal distribution and potential sources of atmospheric microplastic deposition in a semiarid urban environment of Northwest China. Environ. Sci. Pollut. Res. Int..

[28] Jones J.I., Vdovchenko A., Cooling D., Murphy J.F., Arnold A., Pretty J.L., Spencer K.L., Markus A.A., Vethaak A.D., Resmini M. (2020). Systematic Analysis of the Relative Abundance of Polymers Occurring as Microplastics in Freshwaters and Estuaries. Int. J. Environ. Res. Public Health.

[29] Miranda-Peña L., Buitrago-Duque L., Rangel-Buitrago N., Gracia C.A., Arana V.A., Trilleras J. (2023). Geographical heterogeneity and dominant polymer types in microplastic contamination of lentic ecosystems: Implications for methodological standardization and future research. RSC Adv..

[30] Evangelou I., Bucci S., Stohl A. (2026). Atmospheric microplastic emissions from land and ocean. Nature.

[31] Sun Q., Ren S.Y., Ni H.G. (2020). Incidence of microplastics in personal care products: An appreciable part of plastic pollution. Sci. Total Environ..

[32] Lin Q., Pang L., Ngo H.H., Guo W., Zhao S., Liu L., Chen L., Li F. (2023). Occurrence of microplastics in three types of household cleaning products and their estimated emissions into the aquatic environment. Sci. Total Environ..

[33] Zhang S., Wang W., Yan P., Wang J., Yan S., Liu X., Aurangzeib M. (2023). Microplastic migration and distribution in the terrestrial and aquatic environments: A threat to biotic safety. J. Environ. Manag..

[34] Sun P., Liu X., Zhang M., Li Z., Cao C., Shi H., Yang Y., Zhao Y. (2021). Sorption and leaching behaviors between aged MPs and BPA in water: The role of BPA binding modes within plastic matrix. Water Res..

[35] Liu X., Zheng M., Wang L., Ke R., Lou Y., Zhang X., Dong X., Zhang Y. (2022). Corrigendum to “Sorption behaviors of tris-(2,3-dibromopropyl) isocyanurate and hexabromocyclododecanes on polypropylene microplastics” [Mar. Pollut. Bull. 135 (2018) 581–586]. Mar. Pollut. Bull..

[36] Guo X., Wang J. (2019). The chemical behaviors of microplastics in marine environment: A review. Mar. Pollut. Bull..

[37] Thakur B., Singh J., Singh J., Angmo D., Vig A.P. (2023). Biodegradation of different types of microplastics: Molecular mechanism and degradation efficiency. Sci. Total Environ..

[38] Liu L., Xu M., Ye Y., Zhang B. (2022). On the degradation of (micro)plastics: Degradation methods, influencing factors, environmental impacts. Sci. Total Environ..

[39] Yin K., Wang Y., Zhao H., Wang D., Guo M., Mu M., Liu Y., Nie X., Li B., Li J. (2021). A comparative review of microplastics and nanoplastics: Toxicity hazards on digestive, reproductive and nervous system. Sci. Total Environ..

[40] Huang S., Huang X., Bi R., Guo Q., Yu X., Zeng Q., Huang Z., Liu T., Wu H., Chen Y. (2022). Detection and Analysis of Microplastics in Human Sputum. Environ. Sci. Technol..

[41] Ragusa A., Svelato A., Santacroce C., Catalano P., Notarstefano V., Carnevali O., Papa F., Rongioletti M.C.A., Baiocco F., Draghi S. (2021). Plasticenta: First evidence of microplastics in human placenta. Environ. Int..

[42] Leslie H.A., van Velzen M.J.M., Brandsma S.H., Vethaak A.D., Garcia-Vallejo J.J., Lamoree M.H. (2022). Discovery and quantification of plastic particle pollution in human blood. Environ. Int..

[43] Wu D., Feng Y., Wang R., Jiang J., Guan Q., Yang X., Wei H., Xia Y., Luo Y. (2023). Pigment microparticles and microplastics found in human thrombi based on Raman spectral evidence. J. Adv. Res..

[44] Rotchell J.M., Jenner L.C., Chapman E., Bennett R.T., Bolanle I.O., Loubani M., Sadofsky L., Palmer T.M. (2023). Detection of microplastics in human saphenous vein tissue using μFTIR: A pilot study. PLoS ONE.

[45] Ragusa A., Notarstefano V., Svelato A., Belloni A., Gioacchini G., Blondeel C., Zucchelli E., De Luca C., D’Avino S., Gulotta A. (2022). Raman Microspectroscopy Detection and Characterisation of Microplastics in Human Breastmilk. Polymers.

[46] Liu S., Guo J., Liu X., Yang R., Wang H., Sun Y., Chen B., Dong R. (2023). Detection of various microplastics in placentas, meconium, infant feces, breastmilk and infant formula: A pilot prospective study. Sci. Total Environ..

[47] Pauly J.L., Stegmeier S.J., Allaart H.A., Cheney R.T., Zhang P.J., Mayer A.G., Streck R.J. (1998). Inhaled cellulosic and plastic fibers found in human lung tissue. Cancer Epidemiol. Biomark. Prev..

[48] Ibrahim Y.S., Tuan Anuar S., Azmi A.A., Wan Mohd Khalik W.M.A., Lehata S., Hamzah S.R., Ismail D., Ma Z.F., Dzulkarnaen A., Zakaria Z. (2021). Detection of microplastics in human colectomy specimens. JGH Open.

[49] Amato-Lourenço L.F., Carvalho-Oliveira R., Júnior G.R., Dos Santos Galvão L., Ando R.A., Mauad T. (2021). Presence of airborne microplastics in human lung tissue. J. Hazard. Mater..

[50] Jenner L.C., Rotchell J.M., Bennett R.T., Cowen M., Tentzeris V., Sadofsky L.R. (2022). Detection of microplastics in human lung tissue using μFTIR spectroscopy. Sci. Total Environ..

[51] Horvatits T., Tamminga M., Liu B., Sebode M., Carambia A., Fischer L., Püschel K., Huber S., Fischer E.K. (2022). Microplastics detected in cirrhotic liver tissue. eBioMedicine.

[52] Liu S., Liu X., Guo J., Yang R., Wang H., Sun Y., Chen B., Dong R. (2023). The Association Between Microplastics and Microbiota in Placentas and Meconium: The First Evidence in Humans. Environ. Sci. Technol..

[53] Zhu L., Kang Y., Ma M., Wu Z., Zhang L., Hu R., Xu Q., Zhu J., Gu X., An L. (2024). Tissue accumulation of microplastics and potential health risks in human. Sci. Total Environ..

[54] Zhu L., Zhu J., Zuo R., Xu Q., Qian Y., An L. (2023). Identification of microplastics in human placenta using laser direct infrared spectroscopy. Sci. Total Environ..

[55] Massardo S., Verzola D., Alberti S., Caboni C., Santostefano M., Eugenio Verrina E., Angeletti A., Lugani F., Ghiggeri G.M., Bruschi M. (2024). MicroRaman spectroscopy detects the presence of microplastics in human urine and kidney tissue. Environ. Int..

[56] Yun X., Liang L., Tian J., Li N., Chen Z., Zheng Y., Duan S., Zhang L. (2024). Raman-guided exploration of placental microplastic exposure: Unraveling the polymeric tapestry and assessing developmental implications. J. Hazard. Mater..

[57] Lee D.W., Jung J., Park S.A., Lee Y., Kim J., Han C., Kim H.C., Lee J.H., Hong Y.C. (2024). Microplastic particles in human blood and their association with coagulation markers. Sci. Rep..

[58] Sharma G.C., Kumar N., Devrukhkar D., Joshi R.M., Debnath S., Chaturvedi B., Rajotiya S., Mishra S., Sharma A., Singh M. (2026). Human reproductive exposure to microplastics: A multi-technique analytical study of menstrual and amniotic fluids. NanoImpact.

[59] Zhao Q., Zhu L., Weng J., Jin Z., Cao Y., Jiang H., Zhang Z. (2023). Detection and characterization of microplastics in the human testis and semen. Sci. Total Environ..

[60] Gaa P.K., Steele-Dadzie R.K., Boateng L., Intiful F.D., Cobbina S.J., Mogre V. (2026). A Scoping Review of Microplastic Contamination in Infant Foods: Exposure Pathways and Implications for Nutrition and Health of Children. Nutr. Rev..

[61] Ageel H.K., Harrad S., Abdallah M.A. (2024). Microplastics in indoor air from Birmingham, UK: Implications for inhalation exposure. Environ. Pollut..

[62] Mohamed E.F., El-Mekawy A., Abdel-Latif N.M. (2025). Airborne microplastic contamination and health risks in Greater Cairo, Egypt. Environ. Sci. Pollut. Res. Int..

[63] Wright S.L., Kelly F.J. (2017). Plastic and Human Health: A Micro Issue?. Environ. Sci. Technol..

[64] Banerjee A., Shelver W.L. (2021). Micro- and nanoplastic induced cellular toxicity in mammals: A review. Sci. Total Environ..

[65] Liu M., Liu J., Xiong F., Xu K., Pu Y., Huang J., Zhang J., Pu Y., Sun R., Cheng K. (2023). Research advances of microplastics and potential health risks of microplastics on terrestrial higher mammals: A bibliometric analysis and literature review. Environ. Geochem. Health.

[66] Wang X., Deng K., Zhang P., Chen Q., Magnuson J.T., Qiu W., Zhou Y. (2024). Microplastic-mediated new mechanism of liver damage: From the perspective of the gut-liver axis. Sci. Total Environ..

[67] Wei Z., Wang Y., Wang S., Xie J., Han Q., Chen M. (2022). Comparing the effects of polystyrene microplastics exposure on reproduction and fertility in male and female mice. Toxicology.

[68] Xu W., Yuan Y., Tian Y., Cheng C., Chen Y., Zeng L., Yuan Y., Li D., Zheng L., Luo T. (2023). Oral exposure to polystyrene nanoplastics reduced male fertility and even caused male infertility by inducing testicular and sperm toxicities in mice. J. Hazard. Mater..

[69] Zeng L., Zhou C., Xu W., Huang Y., Wang W., Ma Z., Huang J., Li J., Hu L., Xue Y. (2023). The ovarian-related effects of polystyrene nanoplastics on human ovarian granulosa cells and female mice. Ecotoxicol. Environ. Saf..

[70] Lee K.W., Shim W.J., Kwon O.Y., Kang J.H. (2013). Size-dependent effects of micro polystyrene particles in the marine copepod *Tigriopus japonicus*. Environ. Sci. Technol..

[71] Duan Z., Duan X., Zhao S., Wang X., Wang J., Liu Y., Peng Y., Gong Z., Wang L. (2020). Barrier function of zebrafish embryonic chorions against microplastics and nanoplastics and its impact on embryo development. J. Hazard. Mater..

[72] Luo T., Zhang Y., Wang C., Wang X., Zhou J., Shen M., Zhao Y., Fu Z., Jin Y. (2019). Maternal exposure to different sizes of polystyrene microplastics during gestation causes metabolic disorders in their offspring. Environ. Pollut..

[73] Huang W., Mo J., Li J., Wu K. (2024). Exploring developmental toxicity of microplastics and nanoplastics (MNPS): Insights from investigations using zebrafish embryos. Sci. Total Environ..

[74] Budhwar M., Mehra S., Sharma M., Ahsan A.U., Chopra M. (2025). Unveiling micro-nanoplastics (MNPs) induced developmental toxicity, transgenerational transport and associated signaling pathways. J. Hazard. Mater. Adv..

[75] Zhang J., Hu G., Guo H., Yang W., Li X., Ni Y., He M., Ding P., Yu Y. (2025). Amino modifications exacerbate the developmental abnormalities of polystyrene microplastics via mitochondria-mediated apoptosis pathway in zebrafish larvae. Sci. Total Environ..

[76] Tian L., Zhang Y., Chen J., Liu X., Nie H., Li K., Liu H., Lai W., Shi Y., Xi Z. (2024). Effects of nanoplastic exposure during pregnancy and lactation on neurodevelopment of rat offspring. J. Hazard. Mater..

[77] Yu S.P., Chan B.K.K. (2020). Intergenerational microplastics impact the intertidal barnacle *Amphibalanus amphitrite* during the planktonic larval and benthic adult stages. Environ. Pollut..

[78] Rezvanfar M.A., Hodjat M., Abdollahi M. (2016). Growing knowledge of using embryonic stem cells as a novel tool in developmental risk assessment of environmental toxicants. Life Sci..

[79] Sachinidis A., Albrecht W., Nell P., Cherianidou A., Hewitt N.J., Edlund K., Hengstler J.G. (2019). Road Map for Development of Stem Cell-Based Alternative Test Methods. Trends Mol. Med..

[80] Gao T., Sun B., Xu Z., Chen Q., Yang M., Wan Q., Song L., Chen G., Jing C., Zeng E.Y. (2022). Exposure to polystyrene microplastics reduces regeneration and growth in planarians. J. Hazard. Mater..

[81] Liu L., Michowski W., Kolodziejczyk A., Sicinski P. (2019). The cell cycle in stem cell proliferation, pluripotency and differentiation. Nat. Cell Biol..

[82] Zakrzewski W., Dobrzyński M., Szymonowicz M., Rybak Z. (2019). Stem cells: Past, present, and future. Stem Cell Res. Ther..

[83] Rauth S., Karmakar S., Batra S.K., Ponnusamy M.P. (2021). Recent advances in organoid development and applications in disease modeling. Biochim. Biophys. Acta Rev. Cancer.

[84] Hu B., Yin N., Yang R., Liang S., Liang S., Faiola F. (2020). Silver nanoparticles (AgNPs) and AgNO_3_ perturb the specification of human hepatocyte-like cells and cardiomyocytes. Sci. Total Environ..

[85] Chandy M., Obal D., Wu J.C. (2022). Elucidating effects of environmental exposure using human-induced pluripotent stem cell disease modeling. EMBO Mol. Med..

[86] Wang J., Xie L.G., Wu X.F., Zhao Z.G., Lu Y., Sun H.M. (2024). Impact of micro-nano plastics in daily life on human health: Toxicological evaluation from the perspective of normal tissue cells and organoids. Toxicol. Res..

[87] Goodman K.E., Hare J.T., Khamis Z.I., Hua T., Sang Q.A. (2021). Exposure of Human Lung Cells to Polystyrene Microplastics Significantly Retards Cell Proliferation and Triggers Morphological Changes. Chem. Res. Toxicol..

[88] Yang S., Lee S., Lee Y., Cho J.H., Kim S.H., Ha E.S., Jung Y.S., Chung H.Y., Kim M.S., Kim H.S. (2023). Cationic nanoplastic causes mitochondrial dysfunction in neural progenitor cells and impairs hippocampal neurogenesis. Free Radic. Biol. Med..

[89] Zhang M., Zhang R., Kong Y., Li J., Wang G., Wu D., Lan H., Wu M. (2025). Microplastics Exposure Causes the Growth Hormone Resistance on the Stem Cell. J. Biochem. Mol. Toxicol..

[90] Jiang L., Ye Y., Han Y., Wang Q., Lu H., Li J., Qian W., Zeng X., Zhang Z., Zhao Y. (2024). Microplastics dampen the self-renewal of hematopoietic stem cells by disrupting the gut microbiota-hypoxanthine-Wnt axis. Cell Discov..

[91] Yin Z., Zhang Q., Zhu Y., Fu X., Zhong Q., Ou R., Shen H., Mahati S., Li G., Liu Z. (2025). First identification of microplastics in umbilical cord blood and their direct target proteins: A pioneering discovery. Ecotoxicol. Environ. Saf..

[92] Sangkham S., Faikhaw O., Munkong N., Sakunkoo P., Arunlertaree C., Chavali M., Mousazadeh M., Tiwari A. (2022). A review on microplastics and nanoplastics in the environment: Their occurrence, exposure routes, toxic studies, and potential effects on human health. Mar. Pollut. Bull..

[93] Martin-Folgar R., González-Caballero M.C., Torres-Ruiz M., Cañas-Portilla A.I., de Alba González M., Liste I., Morales M. (2024). Molecular effects of polystyrene nanoplastics on human neural stem cells. PLoS ONE.

[94] Zhou B., Wei Y., Chen L., Zhang A., Liang T., Low J.H., Liu Z., He S., Guo Z., Xie J. (2024). Microplastics exposure disrupts nephrogenesis and induces renal toxicity in human iPSC-derived kidney organoids. Environ. Pollut..

[95] Han Y., Zhao J., Huang R., Xia M., Wang D. (2018). Omics-Based Platform for Studying Chemical Toxicity Using Stem Cells. J. Proteome Res..

[96] Płuciennik K., Sicińska P., Misztal W., Bukowska B. (2024). Important Factors Affecting Induction of Cell Death, Oxidative Stress and DNA Damage by Nano- and Microplastic Particles In Vitro. Cells.

[97] Zheng J.H., Li Y.T., Yang S.T., Jia S.Y., Zheng L.W., Wan M. (2025). Silent saboteurs: How microplastics disrupt stem cells and tissue regeneration. World J. Stem Cells.

[98] Bojic S., Falco M.M., Stojkovic P., Ljujic B., Gazdic Jankovic M., Armstrong L., Markovic N., Dopazo J., Lako M., Bauer R. (2020). Platform to study intracellular polystyrene nanoplastic pollution and clinical outcomes. Stem Cells.

[99] Li J., Weng H., Liu S., Li F., Xu K., Wen S., Chen X., Li C., Nie Y., Liao B. (2024). Embryonic exposure of polystyrene nanoplastics affects cardiac development. Sci. Total Environ..

[100] Pan C., Hong R., Wang K., Shi Y., Fan Z., Liu T., Chen H. (2025). Chronic exposure to polystyrene microplastics triggers osteoporosis by breaking the balance of osteoblast and osteoclast differentiation. Toxicology.

[101] Najahi H., Alessio N., Squillaro T., Conti G.O., Ferrante M., Di Bernardo G., Galderisi U., Messaoudi I., Minucci S., Banni M. (2022). Environmental microplastics (EMPs) exposure alter the differentiation potential of mesenchymal stromal cells. Environ. Res..

[102] Park K.Y., Kim M.S., Oh N. (2024). Cytotoxicity of amine-modified polystyrene MPs and NPs on neural stem cells cultured from mouse subventricular zone. Heliyon.

[103] González-Caballero M.C., de Alba González M., Torres-Ruiz M., Iglesias-Hernández P., Zapata V., Terrón M.C., Sachse M., Morales M., Martin-Folgar R., Liste I. (2024). Internalization and toxicity of polystyrene nanoplastics on inmortalized human neural stem cells. Chemosphere.

[104] Guo X., Cheng C., Chen L., Cao C., Li D., Fan R., Wei X. (2023). Metabolomic characteristics in human CD34(+) hematopoietic stem/progenitor cells exposed to polystyrene nanoplastics. Food Chem. Toxicol..

[105] Zhang A., Wang Y., Xue Q., Yao J., Chen L., Feng S., Shao J., Guo Z., Zhou B., Xie J. (2025). Polystyrene microplastics disrupt kidney organoid development via oxidative stress and Bcl-2/Bax/caspase pathway. Chem. Biol. Interact..

[106] Hua T., Kiran S., Li Y., Sang Q.A. (2022). Microplastics exposure affects neural development of human pluripotent stem cell-derived cortical spheroids. J. Hazard. Mater..

[107] Huang F., You H., Tang X., Su Y., Peng H., Li H., Wei Z., Hua J. (2025). Early-life exposure to polypropylene nanoplastics induces neurodevelopmental toxicity in mice and human iPSC-derived cerebral organoids. J. Nanobiotechnol..

[108] Brouwer H., Busch M., Yang S., Venus T., Aalderink G., Crespo J.F.F., Villacorta A., Hernández A., Estrela-Lopis I., Boeren S. (2025). Toxicity of true-to-life microplastics to human iPSC-derived intestinal epithelia correlates to their protein corona composition. J. Hazard. Mater..

[109] Zhang J., Hu H., Zhu Y., Xin X., Jin Y., Zhao Q., Zhang H., Heng D., Ma Z., Chai X. (2025). Polystyrene/polylactic acid microplastics impair transzonal projections and oocyte maturation via gut microbiota-mediated lipoprotein lipase inhibition. J. Hazard. Mater..

[110] Chia S.P.S., Pang J.K.S., Winanto W., Soh B.S. (2025). Nanoplastics induces arrhythmia in human stem-cells derived cardiomyocytes. Ecotoxicol. Environ. Saf..

[111] Ma J., Ladd D.M., Kaval N., Wang H.S. (2025). Toxicity of long term exposure to low dose polystyrene microplastics and nanoplastics in human iPSC-derived cardiomyocytes. Food Chem. Toxicol..

[112] Zhou Y., Wu Q., Li Y., Feng Y., Wang Y., Cheng W. (2023). Low-dose of polystyrene microplastics induce cardiotoxicity in mice and human-originated cardiac organoids. Environ. Int..

[113] Cheng W., Li X., Zhou Y., Yu H., Xie Y., Guo H., Wang H., Li Y., Feng Y., Wang Y. (2022). Polystyrene microplastics induce hepatotoxicity and disrupt lipid metabolism in the liver organoids. Sci. Total Environ..

[114] Wang Y., Zhang A., Liang T., Chen L., Feng S., Zhao Z., Jing Z., Lv J., Xie J., Zhou B. (2025). Polystyrene microplastics induce nephrotoxicity through DDIT4-mediated autophagy and apoptosis. Ecotoxicol. Environ. Saf..

[115] Qin X., Cao M., Peng T., Shan H., Lian W., Yu Y., Shui G., Li R. (2024). Features, Potential Invasion Pathways, and Reproductive Health Risks of Microplastics Detected in Human Uterus. Environ. Sci. Technol..

[116] Shao Y., Li Y., Wang D. (2024). Polylactic acid microplastics cause transgenerational reproductive toxicity associated with activation of insulin and hedgehog ligands in *C. elegans*. Sci. Total Environ..

[117] Manabe S., Haga Y., Tsujino H., Ikuno Y., Idehara W., Hokaku M., Bo Bo Aung P., Asahara H., Higashisaka K., Tsutsumi Y. (2025). Vacuum ultraviolet-induced degradation of polyethylene and polyvinyl chloride micro/nanoplastics enhances their cytotoxicity and lipid peroxidation level. Ecotoxicol. Environ. Saf..

[118] Lawrence M.L., Elhendawi M., Morlock M., Liu W., Liu S., Palakkan A., Seidl L.F., Hohenstein P., Sjögren A.K., Davies J.A. (2022). Human iPSC-derived renal organoids engineered to report oxidative stress can predict drug-induced toxicity. iScience.

[119] Han P., Chen M., Zhang Q., Xie L. (2026). Development and Application of Organoids for Toxicology Studies. J. Appl. Toxicol..

[120] Han W., Cui J., Sun G., Miao X., Pufang Z., Nannan L. (2024). Nano-sized microplastics exposure induces skin cell senescence via triggering the mitochondrial localization of GSDMD. Environ. Pollut..

[121] Cho Y., Seo E.U., Hwang K.S., Kim H., Choi J., Kim H.N. (2024). Evaluation of size-dependent uptake, transport and cytotoxicity of polystyrene microplastic in a blood-brain barrier (BBB) model. Nano Converg..

[122] Liu Y., Wu S., Chen L., Teng X., Shi H., Xue C., Li Z. (2025). Metabolic profiles and protein expression responses of Pacific oyster (*Crassostrea gigas*) to polystyrene microplastic stress. Food Chem..

[123] Yu J., Gu W., Chen L., Wu B. (2023). Comparison of metabolome profiles in zebrafish (*Danio rerio*) intestine induced by polystyrene microplastics with different sizes. Environ. Sci. Pollut. Res. Int..

[124] Wang Q., Wu Y., Zhang W., Shen T., Li H., Wu J., Zhang L., Qin L., Chen R., Gu W. (2022). Lipidomics and transcriptomics insight into impacts of microplastics exposure on hepatic lipid metabolism in mice. Chemosphere.

[125] Fan J., Qu Y., Qu L., Shen D., Liu H., Nie Z. (2025). Oral exposure to PLA microplastics induces time-dependent nanotoxicity via the gut-liver axis. J. Hazard. Mater..

[126] Cheng W., You Y., Chen H., Zhou Y., Feng Y., Wang Y. (2025). Integrated transcriptomics and metabolomics to explore the varied hepatic toxicity induced by aged- and pristine-microplastics: In vivo and human-originated liver organoids-based in vitro study. Environ. Res..

[127] Yang X., Shi J., Shi B., Li J., Xue C., Ma J., Gao X. (2024). Micro- and nano-fibers for organ-on-a-chip: Construction, applications, and prospects. Mater. Today Bio.

[128] Zhang F., Sun M., Zhang J., Xuanyuan T., Yu D., Liu S., Liu W. (2025). An integrated microfluidic pulmonary alveolus system for gradient-controlled investigation of nanoplastic-triggered lung inflammation and injury dynamics. J. Hazard. Mater..

[129] Ciallella H.L., Russo D.P., Sharma S., Li Y., Sloter E., Sweet L., Huang H., Zhu H. (2022). Predicting Prenatal Developmental Toxicity Based On the Combination of Chemical Structures and Biological Data. Environ. Sci. Technol..

[130] Enyoh C.E., Wang Q., Ovuoraye P.E., Maduka T.O. (2022). Toxicity evaluation of microplastics to aquatic organisms through molecular simulations and fractional factorial designs. Chemosphere.

[131] Zhao Y., Zhang S., Liu Y., Li S., Hou J. (2025). Polystyrene modulation of perfluorooctanoic acid toxicity in zebrafish: Transcriptomic and toxicological insights. J. Hazard. Mater..

[132] Silva C.J.M., Machado A.L., Campos D., A M.V.M.S., Pestana J.L.T. (2022). Combined effects of polyethylene microplastics and natural stressors on *Chironomus riparius* life-history traits. Environ. Res..

[133] Yang Y.F., Chen C.Y., Lu T.H., Liao C.M. (2019). Toxicity-based toxicokinetic/toxicodynamic assessment for bioaccumulation of polystyrene microplastics in mice. J. Hazard. Mater..

[134] Planche C., Lievens S., Van der Donck T., Sicard J., Van Der Borght M. (2025). Exposure of black soldier fly larvae to microplastics of various sizes and shapes: Ingestion and egestion dynamics and kinetics. Waste Manag..

[135] Guo X., Wang L., Wang X., Li D., Wang H., Xu H., Liu Y., Kang R., Chen Q., Zheng L. (2024). Discovery and analysis of microplastics in human bone marrow. J. Hazard. Mater..

[136] Rindelaub J.D., Miskelly G.M. (2025). Inhalable microplastics and plastic additives in the indoor air of chemical laboratories. J. Expo. Sci. Environ. Epidemiol..

